# Access to urban green spaces and use of social services and institutional long-term care among older people in Malmö, Sweden: a longitudinal register study

**DOI:** 10.1186/s12877-024-05112-z

**Published:** 2024-06-04

**Authors:** Anna Axmon, Kristoffer Mattisson, Connie Lethin, Agneta Malmgren Fänge, Gunilla Carlsson, Emilie Stroh

**Affiliations:** 1 EPI@LUND (Epidemiology, population research, and infrastructures), Department of Laboratory Medicine, Lund University, Biskopsgatan 9, Lund, SE-223 62 Sweden; 2 Planetary Health, Department of Laboratory Medicine, Lund University, Biskopsgatan 9, Lund, SE- 223 62 Sweden; 3 Department of Health Sciences, Lund University, P.O. Box 117, Lund, SE-22362 Sweden

**Keywords:** Aging, Built environment, Geographical information systems, Health, Neighborhood, Older people, Outdoors, Social services, Urban green

## Abstract

**Background:**

Finding ways to prolong independence in daily life among older people would be beneficial for both individuals and society. Urban green spaces have been found to improve health, but only a few studies have evaluated the association between urban green spaces and independence in daily life. The aim of this study was to assess the long-term effect of urban green spaces on independence in daily life, using social services and support, mobility aids, and relocation to institutional long-term care as proxies, among community dwelling people 65 + years.

**Methods:**

We identified 40 357 people 65 + years living in the city of Malmö, Sweden in 2010. Using geographical information systems (GIS), we determined the amount of urban green spaces (total, public, and quiet) within 300 m of each person’s residence. All three measures were categorized based on their respective percentiles, so that the first quartile represented the 25% with the least access and the fourth quartile the 25% with the most access. In 2015 and 2019, we assessed the outcomes minor assistance (non-personal support), major assistance (personal support), and relocation into institutional long-term care. These three outcome measures were used as proxies for independence in daily life. The effect of amount of urban green spaces in 2010 on the three outcomes in 2015 and 2019, respectively, was assessed by pairwise comparing the three highest quartiles to the lowest.

**Results:**

Compared to the lowest quartile, those in the highest quartile of quiet green spaces in 2010 were less likely to receive minor assistance in both 2015 and 2019. Besides this, there were no indications that any of the measures of urban green space affected independence in daily life at the five- and nine-year follow-up, respectively.

**Conclusion:**

Although urban green spaces are known to have positive impact on health, physical activity, and social cohesion among older people, we found no effect of total, public, or quiet green spaces on independence in daily life. This could possibly be a result of the choice of measures of urban green spaces, including spatial and temporal aspects, an inability to capture important qualitative aspects of the green spaces, or the proxy measures used to assess independence in daily life.

## Background

Growing old is associated with declining mental, cognitive, and physical health. This may to different extents decrease an ageing person’s ability to manage their lives by themselves, i.e., their independence in daily life [1]. This may be reflected in the increasing need for assistance by means of a person, technical devices, or other services. The majority of the older population want to grow old in their own homes – to “age-in-place” [2] – and Sweden has an aging-in-place policy stating that older people should live in their own housing for as long as possible. To achieve this, factors that can sustain independence need to be identified.

Access to urban green spaces has been suggested to be beneficial to mental health [3] and cognitive function [4], as well as to reduce cardiovascular morbidity [5, 6], prevalence of type 2 diabetes [5], fatigue [7], and mortality [8].

Several pathways have been suggested to link urban green spaces to health [9]. These include improved relaxation and restoration, social capital, immune system, and physical activity [10]. Different green environments and green structures might promote aspects of health and wellbeing differently. Private gardens may not only be of service to their owners but might also provide the city or neighborhood with a green aspect that encourages walks or might have beneficial health impacts by their visual abilities to lower stress levels and promote relaxation. Parks and public green spaces do not only enable possibilities for walks but might also provide valuable areas for socializing. Moreover, natural sights may be beneficial to neighborhood social capital of older adults [11], and neighborhood social capital is, in turn, associated with well-being [12]. Furthermore, green spaces may be beneficial, not only due to their own positive effects, but also in that where they exist there is less space for hazardous sources of exposure, such as air pollution from traffic or industries, which has been found to affect the risk of e.g., cardiovascular health [13] and dementia [14]. Supportive structures such as good pathways, benches for rest, and accessible toilets are important features. Also, feeling of safety, cleanliness, serenity, and attended green structures will influence the use and experienced wellbeing in the area [11, 15–17].

A range of tools have been developed to measure independence in daily life [18]. However, most of these rely on assessment for single individuals. To enable objective and more generalizable studies on independence in older people, proxies that can be obtained for larger groups are preferable. In Sweden, people who need support and service to be able to live their lives can obtain this from their municipality. There are several different types of support and services, but as a group they may be divided into those comprising non-personal support (e.g., meals-on-wheels and mobility aids) and those comprising personal support (e.g., help with personal hygiene). Moreover, when receiving support and services in their own homes is no longer enough to manage daily life, there is the option to apply for and be granted relocation to an institutional long-term care (ILTC) facility. Thus, use of social service and support, and relocation to an ILTC facility, might be useful proxies to measure levels of dependence in a large group of older people.

Urban green spaces have been found to be associated with better health and wellbeing. For example, higher amounts of vegetation have been found to be associated with higher levels of physical activity [19], which in turn is associated with better health [20]. So far, only a few studies have investigated the effect of urban green spaces on independence in daily life. In a cross-sectional study, Peng et al. [21] found a non-linear association between residential green and basic activities of daily living (ADL), with higher residential greenness indicating higher ADL performance, among older people. Similar results were found by Zhu et al. [22] at follow-ups up to 12 years. However, both these studies were performed in China, and the results are not necessarily transferable to a Western setting. In a Canadian study, increasing greenness was associated with better self-rated healthy aging at three years follow-up in unadjusted but not adjusted analyses [23]. Moreover, in a previous cross-sectional analyses of older people in Sweden we did not find any association between urban green spaces and use of social services [24]. Thus, more studies, especially longitudinal over different lengths of time, are needed to assess the possible effect of urban green spaces on independence in daily life.

## Methods

The aim of this study was to assess the long-term effect of neighborhood urban green spaces on independence in daily life, using social support and services, mobility aids, and relocation to an ILTC facility as proxies, among people 65 + years living in Malmö, Sweden, in 2010.

### Study context and design

This is a longitudinal, register- and geographical information systems (GIS)-based study among people aged 65 + years living within the city borders of Malmö city in 2010. With approximately 362 000 inhabitants (www.malmo.se), Malmö is the largest city in Skåne, the southernmost region of Sweden. It is the third largest city in Sweden but has a higher population density than the two larger Swedish cities (Gothenburg and Stockholm) [25]. It is situated by Öresund, the strait separating Sweden from Denmark, and is connected to Copenhagen, the Danish capital, by the Öresund bridge. Although Malmö has a long tradition of incorporating parks and recreational areas in the city (www.malmo.se), there is currently a political ambition to continue to densify the city and the municipality [26].

The amount of urban green spaces within 300 m of each person’s residence was determined for 2010, and independence in daily life, measured by use of social services and mobility devices, and relocation to an ILTC facility, was assessed five and nine years later (2015 and 2019, respectively).

### Data sources

- The *National Register of the Total Population* is maintained by Statistics Sweden, the agency responsible for official statistics and other government statistics, and comprises demographic data on all people residing in Sweden.
- The *National Register of Care and Social Services for the Elderly and Persons with Impairments* is maintained by the Swedish National Board of Health and Welfare and comprises monthly data on all social services provided according to the Social Services Act [27], such as meals-on-wheels, help with showering, and grocery shopping. It also includes information about relocation to ILTC facilities.
- In Sweden, people who need a mobility device can have their needs of assistive devices assessed in accordance with current regulations on prescription. If they are deemed eligible, they can borrow such devices from their municipality. This is sometimes associated with a fee. All loans/rentals are registered in the national *SESAM register* using ISO (International organization for standardization) codes together with the date when the person received the device and, when relevant, the date when the device was returned.
- *GIS data on land cover* classifications of vegetation, compiled by Statistics Sweden [28] can be used to quantify the physical amount of public accessible urban green spaces, such as parks, gardens, and recreational areas, within a specified area.
- *Modelled noise levels* from road traffic and railway noise, based on the traffic flows in 2016, were available from the strategic noise mapping conducted in accordance with the END-directive by the municipality of Malmö [29].
- *Demographic Statistical Areas (DeSO)* represent a geographical break-down of Sweden, following county and municipal boundaries. The delimitations can be freely downloaded as open geodata from Statistics Sweden’s website (www.scb.se). A range of sociodemographic variables can then be obtained from the same website.

### Study population

Through the National Register of the Total Population, we identified all people aged 65 + years living in Malmö on December 31st, 2010. From the same register, we collected data on sociodemographic factors. We used DeSO-data to collect information on area socioeconomic standard (SES) measured as the percentage of the population in the DeSO-area with low economic standard (defined as having less than 60% of the national median).

From the 42 407 people identified, we excluded 89 people with supported living, 531 people not living in house or apartment, 18 not categorized as single or cohabiting, and 1 412 living in an ILTC facility in 2010. The remaining 40 357 comprised the study cohort. The mean age in 2010 was 76 years (standard deviation 7.8), and the mean percentage of people with low SES on area-level was 24% (standard deviation 14.8). The majority were born in Sweden (*n* = 31 251, 77%) and living in an apartment (*n* = 32 050, 79%). Slightly more than half were women (*n* = 23 685, 59%) and living alone (*n* = 22 488, 56%).

### Exposure

The percentage of urban green areas within 300 m from the individual’s residence was assessed for 2010, using GIS-data with land cover classifications of vegetation. Three measures of exposure were used:

1. Total urban green spaces - the total ground cover area within the city covered by vegetation, for instance gardens (including private gardens), parks, trees, and other grass surfaces, independent of how they are used.
2. Public urban green spaces – a subset of total urban green spaces consisting of the green areas that are publicly available, with consideration to ownership of the land.
3. Quiet urban green spaces – a subset of public urban green spaces consisting of areas with noise levels below 45 dB(A) LAeq24, based on modelled noise levels from road traffic and railways noise.

For a full description of each measure see Mattisson et al. [30].

### Outcomes

Two data sources were used to assess outcomes for 2015 and 2019, respectively. The National Register of Care and Social Services for the Elderly and Persons with Impairments was used to collect data on social services and support, and relocation to an ILTC facility, whereas the SESAM register was used to identify people with mobility devices provided between January 1st, 2010, and December 31st, 2019. Combining these data, we classified each person into one of the following categories.

1. No assistance: People with no registered use of social service or mobility devices.
2. Minor assistance: People registered as having a safety alarm, meals-on-wheels, or using mobility devices, such as wheelchairs, walkers, walking frames, crutches, or canes; thus, those who only need “non-personal” assistance in their own housing.
3. Major assistance: People who had home care service, in the form of personal care, respite service, or companion service; hence, individual’s dependent on personal assistance to be able to continue to live in their own housing.
4. ILTC: People who were registered as living in or moving to an institutional long-term care home during the year.

Each person was classified based on the highest level of assistance needed. Thus, individuals who were granted living in an ILTC facility during a study year belonged to this group, and this group only, although they might also be registered for minor assistance in terms of e.g., mobility device or safety alarm. For more details, see Stroh et al. [31].

### Statistics

The risk of **relocation to ILTC** was assessed comparing those who relocated to those who did not, the risk of having **major assistance** was assessed comparing those with major assistance to those with no or minor assistance, and the risk of having **minor assistance** was assessed comparing those with minor assistance to those with no assistance. Thus, all outcomes investigated were dichotomous, and we therefore used general linear models with Poisson distribution and log-link to estimate relative risks (RRs) with 95% Confidence Intervals (CIs) for each respective outcome. As the distributions of the three exposure measures (urban green space, public green space, and quiet green space in 2010) were skewed and could not be used as continuous independent variables in the models, we categorized them based on their respective quartiles. Thus, for each exposure, the median and 25th and 75th percentiles were used as cut-off-points. The lowest quartile of exposure was used as reference in the analyses.

The analyses of relocation to an ILTC facility were based on the entire study population, whereas only those not receiving any support in 2010 (*n* = 32 018) were included in the analysis of receiving minor/major assistance in 2015 and 2019.

#### Assistance in 2010

To assess possible differences in independence in 2010 for those who had relocated to an ILTC facility in 2015 and 2019, respectively, we performed analyses including the level of assistance in 2010 and the interaction term between level of assistance and exposure in 2010.

#### Loss to follow-up

Of the 32 018 people who did not have any assistance in 2010, follow-up data were available for 26 227 (82%) in 2015 and for 20 440 (64%) in 2019. Of the 40 357 people not living in ILTC facilities in 2010 (i.e., the whole study population), follow-up data were available for 29 965 (74%) people in 2015 and for 22 013 (55%) in 2019. The main reasons why a person did not contribute with follow-up data were that they were no longer alive, or no longer living in the study area. Increasing age and area SES, having minor or major assistance in 2010, being a man, and living alone were associated with increased risk of being lost to follow-up in 2015 and 2019. Moreover, those living in apartments in 2010 were more likely to be lost to follow-up in 2019. To assess the possible effects of selective loss to follow-up, we assessed interactions between exposure and the relevant variables.

All analyses are adjusted for cohabitation status (living alone/cohabiting), type of housing (apartment/house), sex (men/women), ethnicity (born in Sweden/abroad), age, and area SES.

Data were analyzed with IBM SPSS Statistics 27.0 and 29.0 (IBM Corporation, Armonk, NY, USA). *p*-values less than 0.05 were considered statistically significant.

## Results

### Institutional long-term care

Of those with follow-up data, 1 348 (4%) had moved into an ILTC facility in 2015 and 654 (3%) in 2019. Those living in the second lowest quartile of quiet green spaces in 2010 were less likely to live in ILTC 2015 (Table 1).

Evaluating possible difference in independence in 2010, we found a statistically significant interaction between the level of assistance in 2010 and UGS. In stratified analyses, those with no assistance in 2010 had RR 0.90 (95% CI 0.72–1.13) for Q2 vs. Q1, RR 1.101 (0.81–1.27) for Q3 vs. Q1, and RR 0.94 (0.73–1.22) for Q4 vs. Q1. For those with minor assistance in 2010, the RR for Q2 vs. Q1 was 1.36 (0.93–1.97), for Q3 vs. Q1 it was 1.14 (0.78–1.67), and for Q4 vs. Q1 it was 1.13 (0.73–1.76). Further, those with major assistance in 2010 had RR 0.99 (0.78–1.26) for Q2 vs. Q1, 1.09 (0.85–1.38) for Q3 vs. Q1, and 1.25 (0.95–1.66) for Q4 vs. Q1. No other interactions were found for either year or measure of exposure.

Regarding the possible effects of selective loss to follow-up, we found a statistically significant interaction between moving into ILTC and age for UGS in 2015, but not for any of the other sociodemographic variables (Table 2).

**Table 1:** Institutional long-term care (ILTC) in 2015 and 2019 by exposure quartile in 2010

Exposure	Quartile^1^	ILTC 2015					ILTC 2019				
		n	%	RR	95% CI		n	%	RR	95% CI	
Total urban green spaces	Q1 (0–42%)	325	4	1.00	(ref)		168	3	1.00	(ref)	
	Q2 (42–54%)	367	5	1.01	0.87–1.17		167	3	0.93	0.75–1.15	
	Q3 (54–60%)	381	5	1.08	0.93–1.26		167	3	0.90	0.73–1.12	
	Q4 (>60%)	275	4	1.07	0.90–1.27		152	3	0.98	0.77–1.25	
Public urban green spaces	Q1 (0–19%)	306	4	1.00	(ref)		165	3	1.00	(ref)	
	Q2 (19–29%)	323	4	1.02	0.87–1.19		146	3	0.84	0.67–1.06	
	Q3 (29–38%)	388	5	1.09	0.94–1.27		175	3	0.94	0.76–1.17	
	Q4 (>38%)	331	5	1.06	0.90–1.26		168	3	0.95	0.75–1.20	
Quiet urban green spaces	Q1 (0–0.4%)	307	4	1.00	(ref)		140	2	1.00	(ref)	
	Q2 (0.4–2.6%)	313	4	0.82	0.69–0.96		172	3	1.10	0.87–1.38	
	Q3 (2.6–6%)	340	5	0.87	0.74–1.02		167	3	1.04	0.82–1.31	
	Q4 (>6%)	388	5	0.94	0.80–1.09		175	3	1.08	0.86–1.36	

^1^ Percentage of greenness within 300 m

Footnote: Number and percentage of people in ordinary housing in 2010 and living in institutional long-term care (vs. still in ordinary housing) in 2015 and 2019, respectively, based on category of urban green spaces (quartile) in 2010. Relative risks (RRs) with 95% confidence intervals (CIs) for Q2-Q4 vs. Q1 are adjusted for sex, cohabitation status, type of housing, ethnicity, and area-level socioeconomic status

**Table 2:** *p*-values for interaction between sociodemographic variables and exposure

		Age	Area SES	Type of housing	Cohabitation status	Sex
ILTC 2015	Total urban green spaces	**0.019**	0.503		0.752	0.084
	Public urban green spaces	0.405	0.077		0.866	0.667
	Quiet urban green spaces	0.770	0.353		0.486	0.504
ILTC 2019	Total urban green spaces	0.363	0.653	0.655	0.719	0.063
	Public urban green spaces	0.149	0.822	0.585	0.516	0.141
	Quiet urban green spaces	0.834	0.636	0.636	0.851	0.577
Minor assistance 2015	Total urban green spaces	0.079	0.240		0.552	0.384
	Public urban green spaces	0.050	0.990		0.875	0.373
	Quiet urban green spaces	0.199	0.725		0.661	0.592
Minor assistance 2019	Total urban green spaces	0.383	0.648	0.707	**0.018**	0.178
	Public urban green spaces	0.086	0.514	0.301	0.080	0.118
	Quiet urban green spaces	0.261	0.338	0.974	0.198	0.938
Major assistance 2015	Total urban green spaces	**0.028**	0.369		0.628	0.171
	Public urban green spaces	**0.022**	0.894		0.377	0.516
	Quiet urban green spaces	0.150	0.420		**0.029**	0.956
Major assistance 2019	Total urban green spaces	**0.001**	0.972	**0.036**	0.388	0.071
	Public urban green spaces	0.112	0.499	0.790	0.208	0.645
	Quiet urban green spaces	0.214	0.959	0.322	0.629	0.587

Footnote: *p*-values for interaction between each sociodemographic variable and exposure with respect to different outcomes where the sociodemographic variable is associated with low to follow-up. Bold indicates statistical significance (*p* < 0.05)

### Minor and major assistance

Among those with follow-up data in 2015, 19 207 (73%) still had no assistance, 3 583 (14%) had minor assistance, 2 843 (11%) had major assistance, and 594 (2%) had relocated to an ILTC facility (these comprise a subgroup of people included in the analyses presented above). The corresponding numbers for 2019 were 12 457 (61%) for no assistance, 2 187 (11%) for minor assistance, 5 346 (26%) for major assistance, and 450 (2%) for relocation to an ILTC facility.

People in the highest quartile of quiet urban green spaces in 2010 had lower risk of receiving minor assistance in both 2015 and 2019 (Table 3). No effects were found on any of the measures of urban green spaces in 2010 on major assistance in 2015 or 2019 (Table 4).

When assessing the possible effect of selective loss to follow-up, we found statistically significant interactions between major assistance and age for UGS (in 2015 and 2019) and PGS (in 2015 but not 2019), and between living situation and minor assistance in 2015 for QGS and major assistance in 2019 for UGS, and between cohabiting and major assistance in 2019 for UGS (Table 2).

**Table 3:** Minor assistance (vs. no assistance) in 2015 and 2019

Exposure	Quartile^1^	*Minor assistance 2015*				*Minor assistance 2019*			
		*n*	%	RR	95% CI	*n*	%	RR	95% CI
Total urban green spaces	Q1 (0–42%)	856	15	1.00	(ref)	553	15	1.00	(ref)
	Q2 (42–54%)	913	17	1.01	0.92–1.11	488	14	0.89	0.79–1.01
	Q3 (54–60%)	929	17	1.02	0.93–1.12	563	16	1.03	0.92–1.17
	Q4 (> 60%)	885	14	1.07	0.97–1.19	583	14	1.06	0.93–1.21
Public urban green spaces	Q1 (0–19%)	835	14	1.00	(ref)	504	13	1.00	(ref)
	Q2 (19–29%)	825	14	0.95	0.86–1.05	508	14	0.97	0.86–1.10
	Q3 (29–38%)	910	16	1.00	0.91–1.10	523	15	0.98	0.86–1.10
	Q4 (> 38%)	1013	19	1.10	0.99–1.21	652	19	1.05	0.92–1.20
Quiet urban green spaces	Q1 (0-0.4%)	906	15	1.00	(ref)	584	15	1.00	(ref)
	Q2 (0.4–2.6%)	891	15	0.95	0.86–1.04	527	14	0.91	0.81–1.03
	Q3 (2.6-6%)	937	17	0.99	0.90–1.09	575	16	0.95	0.84–1.07
	Q4 (> 6%)	849	16	0.91	0.82-1.00	501	15	0.88	0.77–0.99

^1^ Percentage of greenness within 300 m

Footnote: Number and percentage of people with no assistance in 2010 and minor assistance (vs. no assistance) in 2015 and 2019, respectively, based on category of urban green spaces (quartile) in 2010. Relative risks (RRs) with 95% confidence intervals (CIs) for Q2-Q4 vs. Q1 are adjusted for sex, cohabitation status, type of housing, ethnicity, and area-level socioeconomic status

**Table 4:** Major assistance (vs. no/minor assistance) in 2015 and 2019

Exposure	Quartile^1^	*Major assistance 2015*				*Major assistance 2019*			
		*n*	%	RR	95% CI	*n*	%	RR	95% CI
Total urban green spaces	Q1 (0–42%)	665	10	1.00	(ref)	1268	26	1.00	(ref)
	Q2 (42–54%)	745	12	1.04	0.93–1.15	1356	29	1.02	0.95–1.10
	Q3 (54–60%)	765	12	1.05	0.95–1.17	1451	30	1.04	0.97–1.13
	Q4 (> 60%)	668	10	1.07	0.96–1.21	1271	23	1.02	0.94–1.11
Public urban green spaces	Q1 (0–19%)	673	10	1.00	(ref)	1293	25	1.00	(ref)
	Q2 (19–29%)	700	11	0.99	0.89–1.10	1328	26	0.98	0.91–1.06
	Q3 (29–38%)	753	12	1.00	0.90–1.11	1419	28	1.00	0.93–1.08
	Q4 (> 38%)	717	12	0.97	0.87–1.09	1306	27	1.00	0.92–1.09
Quiet urban green spaces	Q1 (0-0.4%)	663	10	1.00	(ref)	1301	25	1.00	(ref)
	Q2 (0.4–2.6%)	738	11	1.00	0.90–1.12	1389	27	0.98	0.91–1.06
	Q3 (2.6-6%)	698	11	0.94	0.85–1.06	1304	26	0.93	0.86–1.01
	Q4 (> 6%)	744	12	1.00	0.89–1.11	1352	29	0.98	0.90–1.06

^1^ Percentage of greenness within 300 m

Footnote: Number and percentage of people with no assistance in 2010 and major assistance (vs. no or minor assistance) in 2015 and 2019, respectively based on category of urban green spaces (quartile) in 2010. Relative risks (RRs) with 95% confidence intervals (CIs) for Q2-Q4 vs. Q1 are adjusted for sex, cohabitation status, type of housing, ethnicity, and area-level socioeconomic status

## Discussion

In this study, we found no effect of urban green spaces on increased independence in daily life, using social services and support, mobility devices, and relocation to an ILTC facility as proxies, five and nine years later.

This study is based on different measures of urban green spaces (exposure) and proxies for independence in daily life (outcome). As a measure of *total urban green*, we used the percentage of urban green spaces within 300 m of the individual’s residences. This includes publicly available green spaces as well as private gardens and other green spaces that are not physically accessible but still viewable. Previous studies have found that the latter, i.e., viewing green without being physically in a green space, may reduce stress and induce more positive emotional states (the psycho-physiological stress reduction theory) [32, 33] as well as improve self-rated health [34] and performance in cognitively demanding tasks (attention restoration theory) [35].

A potential weakness of the present study is the large loss to follow-up, at 26% in 2015 and 45% in 2019. Considering the age groups studied, the number of people lost to follow-up is to be expected. However, the loss was not completely random, as e.g., men and people living alone were more likely to be lost to follow-up. If the associations between exposures and outcomes differed between levels of these variables, e.g., between men and women, this could introduce bias. Therefore, for those sociodemographic variables that differed between those included and those lost to follow-up, we assessed possible interaction (i.e., if the associations between exposure and outcomes differed between levels). This was found only for a few combinations of exposure, outcome, and sociodemographic variable. Thus, we do not believe that selection of certain groups of people into the two follow-ups has caused any major bias in the results.

This study used the measure *publicly available urban green space*, e.g., parks and other types of recreational areas. We believe this is a good proxy for possibilities to use and access urban green spaces for the city population in general. However, it does not consider the quality aspect of the urban green spaces, such as perceived cleanliness, safety, noise level, supportive structures such as park benches and available toilets, or the general attractive green structures of the park. Such quality aspects and supportive structures are of great importance when it comes to use of green areas as well as their perceived health benefits [34, 36]. Therefore, we also included a measure based on amount of urban green spaces within 300 m from the residency with a modelled noise level below 45 dB(A) as a proxy for available *quiet urban green space*.

We found no associations between the selected measures of access to urban green spaces and the proxies for independence in daily life. This could possibly be explained by choice of measures of urban green spaces. For example, we only used one measure which considered quality aspects (quiet urban green spaces), while other quality aspects, such as cleanliness, feeling of security, and available supportive structures might be of even greater importance when it comes to promoting use of these urban green spaces for old people [11, 16, 17].

Another possible explanation may be that we used exposure to urban green spaces at a particular time, i.e., in 2010. Thus, we did not consider possible changes in exposure over time, or the effect of aggregated exposure. We have previously found that current exposure is not related to use of social services [24]. In the present study, the aim was to determine if long-term associations were more relevant. It is possible that another measure of exposure, aggregating the exposure over longer periods of time, and taking change of residence into account, would be more appropriate.

The results might also be related to the choice of proxy for independence, i.e., using social services and walking aids to categorize people as needing minor or major assistance. This was based on the assumption that the need for assistance with instrumental activities, such as transportation and cleaning, occurs before the need for assistance with personal activities, such as eating and drinking, arises [37]. However, that a person does not have support to perform a certain activity does not necessarily imply that they can perform that activity themselves but could also be because they have no desire to perform that activity or have help from a spouse or an informal caregiver.

Social services and support are provided after application from the older person themselves and approval from the municipality. This implies that receiving service and support do not equal needing such support. Indeed, several sociodemographic factors have been associated with receiving support [31]. Individuals not receiving support and service from the municipality may be provided for by informal caregivers which might be a spouse, relative, or friend. Moreover, some people needing – or wanting – support with tasks in their daily lives may opt to pay for such services from other providers than the municipality. For example, grocery shopping may be done online with home delivery, and mobility devices may be purchased from stores rather than borrowed from the municipality. In Sweden when you hire a person or company to do household chores that do not require expert knowledge, such as cleaning or mowing the lawn, you can apply for tax reduction based on half the cost of the service provided, so called RUT tax reductions. The use of RUT is skewed towards high income households, and about half of the people using RUT are 57 + years [38]. Thus, there might be a misclassification from minor and major assistance to no assistance that is affected by socioeconomic status. Another weakness in our choice of outcome measures may be that there are no strict rules and guidelines for granting support, meaning that the discrepancy between need and reception may vary between municipalities. However, as we included only one municipality, this should not pose a problem in the present study. Summarizing, receiving social services may not be a proper proxy for independence in daily life, which may be a contributing factor for failing to find an effect of urban green spaces. Even so, the use of register data to assess independence in daily life has several advantages. First, it allows for inclusion of a large number of people. This would not be possible if data had to be collected separately for each single individual, e.g., by interviews. Also, the use of register data ensures inclusion of people and collection of information without selection bias.

## Conclusions

Although urban green spaces are known to have positive effects on health, physical activity, and social cohesion among older people, we found no effect of total, public, or quiet urban green spaces on independence in daily life. This could possibly be a result of the choice of measures of urban green spaces, or the proxy measures used to assess independence in daily life. Future studies should consider a larger variety of measures of green space and, if possible, try to incorporate objective measures of the green qualities in these areas as well as supportive structures. Moreover, future studies should also investigate whether social services indeed can be used to estimate independence in daily life, and if so, which services – by themselves or in combination with other services and factors – are the best proxies.

## Data Availability

The datasets generated and/or analyzed during the current study are not publicly available due to legal reasons but are available from the corresponding author on reasonable request and upon secrecy review according to Swedish law.

## Abbreviations

ADL: Activities of daily living
CI: Confidence interval
DeSO: Demographical statistical area
GIS: Geographical information systems
ILTC: Institutional long-term care
ISO: International organization for standardization
Q: Quartile
RR: Relative risk
SES: Socioeconomic status
WHO: World Health Organization
