# Correction: Access to urban green spaces and use of social services and institutional long-term care among older people in Malmö, Sweden: a longitudinal register study

**DOI:** 10.1186/s12877-024-05279-5

**Published:** 2024-08-20

**Authors:** Anna Axmon, Kristoffer Mattisson, Connie Lethin, Agneta Malmgren Fänge, Gunilla Carlsson, Emilie Stroh

**Affiliations:** 1 EPI@LUND (Epidemiology, population research, and infrastructures), Department of Laboratory Medicine, Lund University, Biskopsgatan 9, Lund, SE-223 62 Sweden; 2 Planetary Health, Department of Laboratory Medicine, Lund University, Biskopsgatan 9, Lund, SE-223 62 Sweden; 3 Department of Health Sciences, Lund University, P.O. Box 117, Lund, SE-22362 Sweden

**Correction: *BMC Geriatr***,**24, 489 (2024)**

10.1186/s12877-024-05112-z

Following publication of the original article [1], the authors reported printing errors in Table 1. The correct table is given below.

**Table 1:** Institutional long-term care (ILTC) in 2015 and 2019 by exposure quartile in 2010

Exposure	Quartile^1^	ILTC 2015				ILTC 2019			
		n	%	RR	95% CI	n	%	RR	95% CI
Total urban green spaces	Q1 (0-42%)	325	4	1.00	(ref)	168	3	1.00	(ref)
	Q2 (42-54%)	367	5	1.01	0.87-1.17	167	3	0.93	0.75-1.15
	Q3 (54-60%)	381	5	1.08	0.93-1.26	167	3	0.90	0.73-1.12
	Q4 (>60%)	275	4	1.07	0.90-1.27	152	3	0.98	0.77-1.25
Public urban green spaces	Q1 (0-19%)	306	4	1.00	(ref)	165	3	1.00	(ref)
	Q2 (19-29%)	323	4	1.02	0.87-1.19	146	3	0.84	0.67-1.06
	Q3 (29-38%)	388	5	1.09	0.94-1.27	175	3	0.94	0.76-1.17
	Q4 (>38%)	331	5	1.06	0.90-1.26	168	3	0.95	0.75-1.20
Quiet urban green spaces	Q1 (0-0.4%)	307	4	1.00	(ref)	140	2	1.00	(ref)
	Q2 (0.4-2.6%)	313	4	0.82	0.69-0.96	172	3	1.10	0.87-1.38
	Q3 (2.6-6%)	340	5	0.87	0.74-1.02	167	3	1.04	0.82-1.31
	Q4 (>6%)	388	5	0.94	0.80-1.09	175	3	1.08	0.86-1.36

^1^ Percentage of greenness within 300 m

Footnote: Number and percentage of people in ordinary housing in 2010 and living in institutional long-term care (vs. still in ordinary housing) in 2015 and 2019, respectively, based on category of urban green spaces (quartile) in 2010. Relative risks (RRs) with 95% confidence intervals (CIs) for Q2-Q4 vs. Q1 are adjusted for sex, cohabitation status, type of housing, ethnicity, and area-level socioeconomic status

The original article [1] has been corrected.
